# Quasilinear quantum magnetoresistance in pressure-induced nonsymmorphic superconductor chromium arsenide

**DOI:** 10.1038/ncomms15358

**Published:** 2017-06-05

**Authors:** Q. Niu, W. C. Yu, K. Y. Yip, Z. L. Lim, H. Kotegawa, E. Matsuoka, H. Sugawara, H. Tou, Y. Yanase, Swee K. Goh

**Affiliations:** 1 Department of Physics, The Chinese University of Hong Kong, Shatin, New Territories, Hong Kong 999077, China; 2 Department of Physics, Kobe University, Kobe 658-8530, Japan; 3 Department of Physics, Kyoto University, Kyoto 606-8502, Japan

## Abstract

In conventional metals, modification of electron trajectories under magnetic field gives rise to a magnetoresistance that varies quadratically at low field, followed by a saturation at high field for closed orbits on the Fermi surface. Deviations from the conventional behaviour, for example, the observation of a linear magnetoresistance, or a non-saturating magnetoresistance, have been attributed to exotic electron scattering mechanisms. Recently, linear magnetoresistance has been observed in many Dirac materials, in which the electron–electron correlation is relatively weak. The strongly correlated helimagnet CrAs undergoes a quantum phase transition to a nonmagnetic superconductor under pressure. Here we observe, near the magnetic instability, a large and non-saturating quasilinear magnetoresistance from the upper critical field to 14 T at low temperatures. We show that the quasilinear magnetoresistance may arise from an intricate interplay between a nontrivial band crossing protected by nonsymmorphic crystal symmetry and strong magnetic fluctuations.

## Abstract (web summary)

The electronic structure of the helimagnet CrAs is unusual due to its nonsymmorphic crystal symmetry. Here, the authors observe quasilinear magnetoresistance close to a pressure-driven superconducting transition, which may arise from the interaction of the band structure and magnetic fluctuations.

Linear magnetoresistance (MR) is a fascinating material property that can be harnessed for magnetic-field sensing owing to its simple field-to-resistance conversion and its equal sensitivity across the entire field range. As such, the mechanisms underpining the linear MR continue to attract interest. Linear MR is most commonly observed in systems with a relatively weak electron–electron correlation and comparatively low carrier density, such as doped semiconductors^1^,^2^,^3^ and topological semimetals^4^,^5^,^6^,^7^. The observation of a linear MR in strongly correlated electron systems has been a rare occurence. A notable case of a striking linear MR is found in BaFe_2_(As_1−*x*_P_*x*_)_2_ near the antiferromagnetic quantum critical point^8^, where non-Fermi liquid behaviour in the temperature (*T*) dependence of the resistivity (*ρ*) has also been observed^9^, leading to a phenomenological model to describe the electrical transport with a scattering rate that is proportional to the quadrature sum of magnetic field and temperature^8^. Signature of linear MR has also been found in some heavy fermion compounds such as UPt_3_ and CeCu_6_ (refs 10, 11), although the conventional quadratic component or the negative MR was found to coexist with the linear component in these systems. To develop the concept further, it is important to study other strongly correlated electron systems near magnetic instability which exhibit clear linear MR to expand the material base for detailed investigations.

CrAs undergoes a strong first-order transition at *T*_N_=265 K into an antiferromagnetic state, in which the spins assume a double helical structure (winding axis || *c* axis)^12^,^13^. This antiferromagnetic state can be completely suppressed by applying 7 kbar (refs 14, 15), or by substituting 5% of phosphorous for arsenic, that is, CrAs_0.95_P_0.05_ (ref. 16). The magnetic behaviour of phosphorous-substituted samples is similar to that of CrAs under high pressure, and hence phosphorous acts as the chemical pressure analogous to other systems such as BaFe_2_(As_1−*x*_P_*x*_)_2_ (ref. 17). Concomitant with the suppression of the helimagnetic state, superconductivity can be induced in CrAs by pressure. The superconducting transition temperature (*T*_c_) takes a dome-shaped pressure dependence, with a maximum *T*_c_≈2.17 K at 10 kbar (refs 14, 15). The temperature–pressure (*T–p*) phase diagram constructed is reminiscent of those constructed for many quantum critical systems, for example, refs 18, 19, 20, 21, 22, except that the magnetic transition at ambient pressure is strongly first order, although the first-order signature is significantly weakened at elevated pressures^14^,^15^. Nevertheless, nuclear quadrupole resonance detected substantial magnetic fluctuations in the paramagnetic state^23^. In the phosphorous-substituted series, superconductivity has not been observed due to the strong disorder introduced by phosphorous substitution, which is consistent with the unconventional nature of superconductivity^23^.

We have measured the MR of CrAs down to 14 mK in representative parts of the *T*–*p* phase diagram. The space group of CrAs, *Pnma*, includes nonsymmorphic glide and screw symmetries, which involve half translation. We will show that the nonsymmorphic symmetry of the crystal structure plays an essential role on the realization of a particular band structure, leading to a nontrivial band crossing on the Brillouin zone face protected by nonsymmorphic symmetry. At 14 T, the MRs are positive, sizeable and non-saturating. Furthermore, distinct quasilinear MRs are observed in the vicinity of the magnetic instability, which we suggest is the result of an intricate interplay between having this particular band structure and the proximity to magnetic instability.

## Results

### *T*–*p* phase diagram and MR

**Figure 1:**
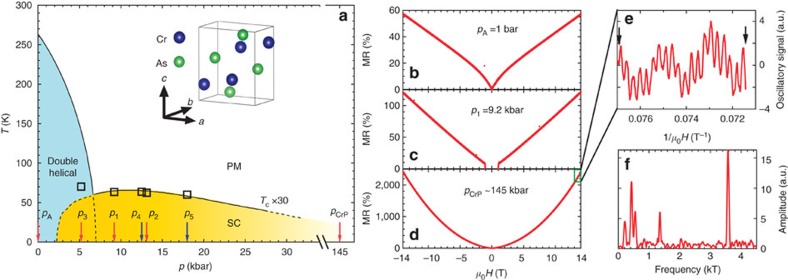
Transverse magnetoresistance in pressure-tuned CrAs. (**a**) Schematic temperature–pressure phase diagram showing the helimagnetic region, the superconducting region (SC) and the paramagnetic region (PM), constructed using the data in ref. 14. The area bounded by dashed lines indicates the region with coexistence of magnetism and superconductivity. The open squares denote the experimental data of the current work, which are consistent with the previously published data^14^,^15^. For clarity, the values of *T*_c_ are multiplied by 30. The position of CrP is determined by taking into account the chemical pressure effect of P on CrAs. The crystal structure of CrAs, prepared using VESTA^42^, is shown in the inset. (**b**–**d**) Magnetoresistance, defined as MR=[*ρ*(*H*)−*ρ*(0)]/*ρ*(0) × 100%, from −14 to 14 T at *p*_A_, *p*_1_, and *p*_CrP_, collected at 25.7, 15.5 and 16.2 mK, respectively. For the data collected in the superconducting state, the magnetoresistance is calculated using a zero-field resistivity *ρ*(0) estimated from a smooth extrapolation of the data above *H*_c2_. (**e**) High-field magnetoresistance in CrP, shown as the green rectangle in **d**, with steep background subtracted. The left and the right arrows are at 13 and 14 T, respectively. Clear Shubnikov–de Haas oscillations can be seen. (**f**) Fast Fourier transform of the oscillatory signal in **e** from 8 to 14 T.

Figure 1a shows the temperature–pressure phase diagram of CrAs. Three crystals were used for this study, namely CrAs at ambient pressure (*p*_A_), CrP at ambient pressure (*p*_CrP_) and CrAs under pressure (*p*_*i*_'s with *i* indicates the sequence of the measurement). Both CrAs and CrP crystallize in an orthorhombic structure with space group *Pnma* (inset to Fig. 1a)^12^. Using the *x*-dependence of the unit cell volume in CrAs_1−*x*_P_*x*_ (ref. 24), we estimate that Δ*x*=1 corresponds to Δ*p*≈145 kbar. Therefore, we place CrP at the location of *p*=145 kbar relative to CrAs. CrP has an excellent purity with a residual resistance ratio (RRR) of 455, in stark contrast to members of phosphorous-substituted CrAs. For example, RRR≈5 for CrAs_0.95_P_0.05_. On the other hand, the RRR of the pressurized CrAs is far larger, for example, RRR=194 at *p*_2_, provided that care is taken not to thermally cycle the crystal through the first-order line (see below). Figure 1b–d show the MR, defined as MR=
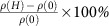
, at the lowest attainable temperature of each run in the helimagnetic state (*p*_A_), nonmagnetic superconducting state (*p*_1_=9.2 kbar) and the paramagnetic non-superconducting state (*p*_CrP_), respectively. All traces exhibit a large and non-saturating MR at 14 T. In the helimagnetic state (*p*_A_), the field dependence of the MR is sublinear at low field with a seemingly absence of the *H*^2^ region. At *p*_1_, the MR is quasilinear from the upper critical field (*H*_c2_) to 14 T. Finally, at *p*_CrP_, the MR is conventional and follows a *H*^2^ variation from 0 to 14 T. The CrAs system thus provides a useful platform for accessing different MR behaviours by tuning the unit cell volume. All these traces are clearly an even function of *H*, indicating a negligible contamination from the Hall component. Therefore, the data presented here are truly the transverse MR and hence only the positive *H* portion of the data are plotted in the following.

**Figure 2:**
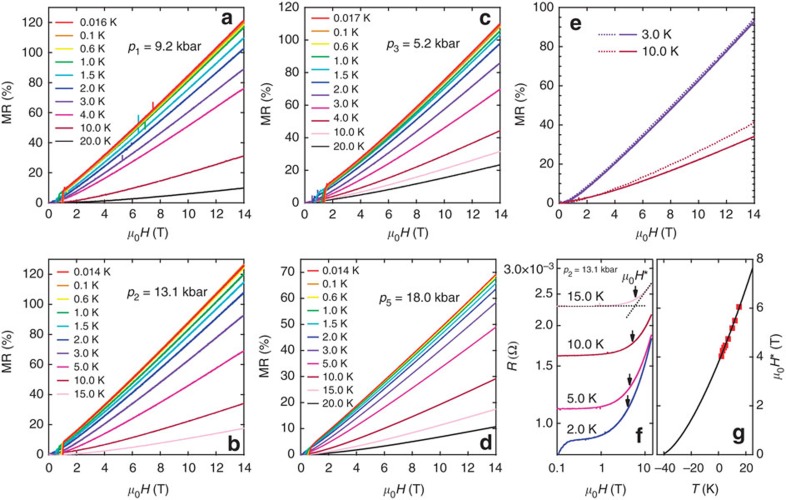
Magnetoresistance of CrAs under pressure. (**a**–**d**) Magnetoresistance of CrAs over a wide temperature range at *p*_1_, *p*_2_, *p*_3_ and *p*_5_. The spikes at some traces are experimental artefacts. (**e**) Magnetoresistance at *p*_2_ (solid lines) and *p*_4_ (dashed lines). The traces at *p*_4_ are multiplied by 1.62, which is the ratio RRR(*p*_2_)/RRR(*p*_4_). (**f**) The field dependence of the resistance at *p*_2_ on logarithmic scales, showing the determination of the crossing over field *μ*_0_H*. (**g**) The temperature dependence of *μ*_0_H* (squares) at *p*_2_, which can be described by *μ*_0_H*=*a*(*T*+*b*)^3/2^ (solid line).

Figure 2a–d show the MR collected over a wide temperature range at pressures where the ground state is superconducting. At these pressures, the MR is quasilinear at the lowest temperature, and it reaches at least 70% at 14 T. For *p*_1_ and *p*_2_, the single crystal was directly pressurized to the required pressure values at room temperature before cooling. MR(14 T) at the lowest temperature for *p*_1_ and *p*_2_ are larger than 110%, and it decreases with increasing temperature. To investigate the possibility of a disorder-induced linear MR behaviour, we take advantage of the strong magnetostriction effect associated with the first-order transition into the helimagnetic state^12^. Empirically, it is known that on warming up across the first-order line, the crystal can crack. Therefore, we reduced the pressure to 5.2 kbar (*p*_3_) and performed a thermal cycle, where the first-order transition exists but much weaker than the ambient pressure case (*p*_A_)^12^,^14^,^15^, with the aim of inducing microcracks that are not completely detrimental to measurements. Indeed, at *p*_4_ and *p*_5_, which are the runs after *p*_3_, RRR decreases to 120 and 96, respectively. Interestingly, although MR(14 T) decreases significantly, the curvature of the MR remains quasilinear. In Fig. 2e, we compare the MR at 3 and 10 K for *p*_2_ and *p*_4_, since these pressure values are very close. We find that the MRs at *p*_4_ can be satisfactorily scaled to the curves at *p*_2_ using the ratio RRR(*p*_2_)/RRR(*p*_4_)≅1.62. This exercise works extremely well for the data sets at 3 K. Therefore, the functional form of the MR is robust in this system. Although it is not possible to completely rule out linear MR mechanisms that assume sample inhomogeneity^2^,^25^, we note that our samples are high-quality single crystals: RRR and the residual resistivity at *p*_2_ are 194 and 0.83 μΩ cm, respectively (Supplementary Note 2). These observations suggest that disorder, particularly spatial inhomogeneity associated with the first-order transition, is not expected to play a dominant role.

### Band structure and the nonsymmorphic symmetry

**Figure 3:**
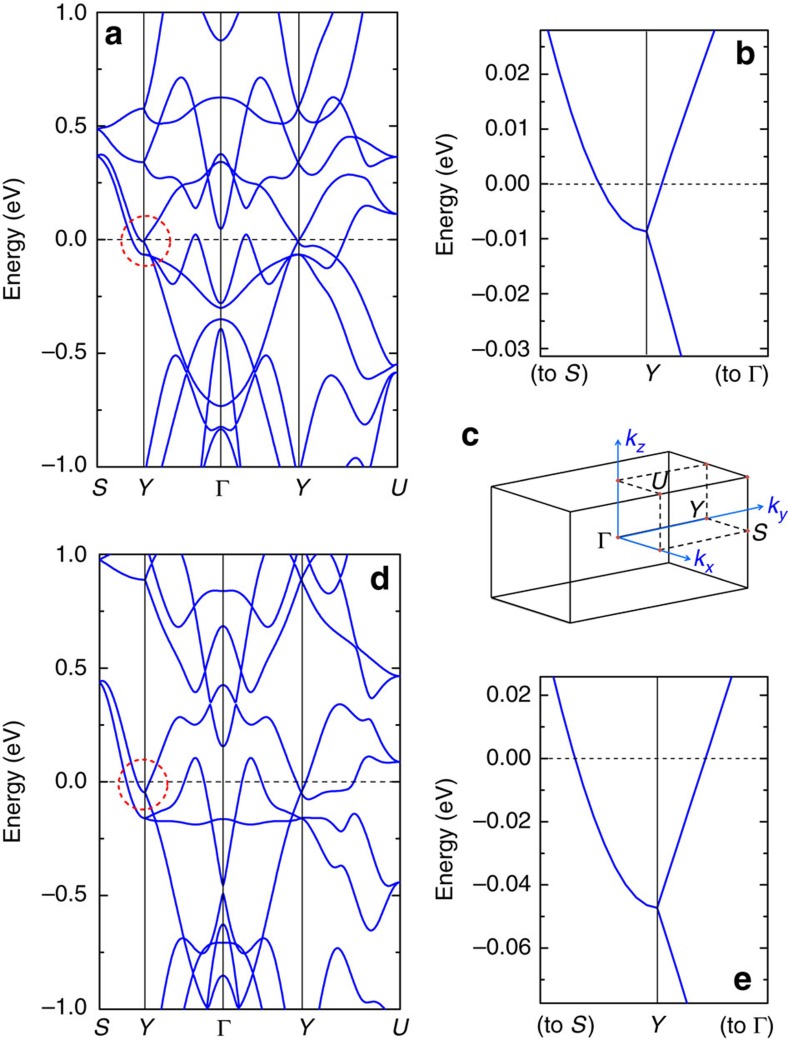
Comparison of the band structure of CrAs at 14.3 kbar and CrP. (**a**,**b**) Dispersion relation along several high-symmetry directions for CrAs at 14.3 kbar. An expanded view of the band structure near *E*_F_ around *Y* (indicated by the circle) is shown, where a band crossing is found ∼8.6 meV below the Fermi energy. The dispersion is linear along *Y*–Γ and parabolic along *Y*–*S*. (**c**) The first Brillouin zone of CrAs/CrP with the relevant high-symmetry points labelled. (**d**,**e**) Dispersion relation for CrP. A similar band structure is found in CrP near *Y*, but with a crossing point located at ∼47 meV below *E*_F_ instead.

Figure 3a shows the band structure of CrAs at 14.3 kbar, calculated using the experimental lattice parameters^24^. At the *Y* point, a band crossing is found at ∼8.6 meV below *E*_F_ along the *k*_*y*_ direction, giving rise to a linear energy–momentum dispersion with a slope *ħc*. Along the *Y*–*S* direction, which is parallel to Γ–*X* (Fig. 3c), the dispersion is quadratic, parametrized by *m*. The crossing point at *Y* is thus analogous to the semi-Dirac point discussed in the literature^26^,^27^, except that the parabolic branches are degenerate in our case. Such a hybrid dispersion relation is a result of the nonsymmorphic symmetry of the space group *Pnma*. The two bands crossing at the *Y* point are degenerate along the *k*_*x*_ direction (*Y*–*S* line on the Brillouin zone face), as the nonsymmorphic symmetry protects the degeneracy. Thus, the band crossing has to occur on the *Y*–*S* line on which the linear term in *k*_*x*_ is prohibited and the usual quadratic dispersion appears (see Supplementary Note 4 for theoretical proof). A similar band crossing at the *Y* point is obtained for CrP, with the crossing point located at ∼47 meV below *E*_F_, which is much deeper compared with CrAs at 14.3 kbar (Fig. 3e). Although a similar band crossing protected by another nonsymmorphic space group *P*4/*nmm* was reported in ZrSiS (ref. 28), the crossing point appears 500 meV below *E*_F_. This is to be contrasted with the present case where the crossing point is much closer to *E*_F_, and the separation between the crossing point and *E*_F_ is tunable by applied pressure (compare Fig. 3b,e).

### Landau levels associated with the hybrid band structure

The close proximity of the crossing point to *E*_F_ implies a small Fermi pocket, which contains neither conventional electrons nor Dirac electrons. In the *k*_*x*_–*k*_*y*_ plane, the dispersion relation around the *Y* point near *E*_F_ can be written as 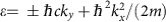, where (*k*_*x*_, *k*_*y*_) is the wavevector measured from *Y*. Combining with the quantization condition *S*(*ɛ*)=2*π*(*n*+*γ*)*eμ*_0_*H*/*ħ* within the semiclassical approach, the Landau level spacing is proportional to *H*^2/3^ when *H* || *k*_*z*_, and *γ*∈[0, 1] is a phase factor that can not be determined from the semiclassical approach. According to Abrikosov's theory, a Fermi surface sheet will contribute to a linear quantum MR if all carriers occupy the lowest Landau level^29^,^30^. This is the extreme quantum limit, and it requires the band extrema to be very near the Fermi energy (*E*_F_). It is a straightforward exercise to calculate the characteristic field *μ*_0_H*, above which all carriers in this pocket are in the lowest Landau level:


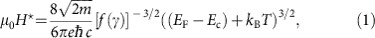


where *E*_c_ is the energy of the crossing point, and *f*(*γ*)=(1+*γ*)^2/3^−*γ*^2/3^ is a dimensionless quantity. Since this pocket has an anisotropy 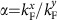 in the *k*_*x*_–*k*_*y*_ plane, we get another constraint *m*=*α*^2^(*E*_F_−*E*_c_)/(2*c*^2^).

Following Narayanan *et al*.^6^, we plot log*R* against log(*μ*_0_*H*) in Fig. 2f, which clearly reveals how the low-field MR transforms into the linear region above a crossover field. Given the absence of quantum oscillations in our high-quality sample, which we will discuss later, it is tempting to assign the crossover field as *μ*_0_H*. That is to say, we speculate that the linear MR in pressurized CrAs arises from the Fermi surface pocket at the *Y* point, which reaches the extreme quantum limit above *μ*_0_H*. The *T*-dependence of *μ*_0_H* at *p*_2_=13.1 kbar, which is the closest to the pressure value of the calculated band structure, can be satisfactorily described by *μ*_0_H*=*a*(*T*+*b*)^3/2^ (see Supplementary Note 1 for other pressures). (*E*_F_−*E*_c_) can be instantly determined from *b* to be 3.7 meV. If the band structure displayed in Fig. 3b is rigidly shifted so that (*E*_F_−*E*_c_)=3.7 meV, we obtain *α*=4.7. From the fitting parameter *a*, we can estimate that *c* ranges between 10.5 × 10^4^ and 15.6 × 10^4^ m s^−1^ when *γ* goes from 0 to 1. This is in satisfactory agreement with *c*=9.9 × 10^4^ m s^−1^ extracted from the band structure calculation at 14.3 kbar. Note that the band shifting does not affect *c*, hence the comparison between experimental data and the calculated slope *ħc* is particularly meaningful. Therefore, it is conceivable that the MR in the pressurized CrAs is governed by the tiny Fermi pocket at *Y*, which reaches the extreme quantum limit above *μ*_0_H*.

For a multiband system like CrAs, the applicability of Abrikosov's theory requires a careful justification. In fact, in his own paper^30^, Abrikosov provided a mechanism to show how the theory can be applied when a big Fermi surface coexists with a small Fermi pocket. Following the same line of thought, we propose that in the pressurized CrAs, the Fermi pocket around *Y* dominates the MR. Along the Γ–*Y* direction, the effective mass is essentially zero because of the linear dispersion. Along the *Y*–*S* direction, *m* ranges between 0.66–0.30 *m*_*e*_ for *γ* between 0 and 1. Therefore, the mobility is high and this pocket can dominate the MR. In the vicinity of the magnetic instability, magnetic fluctuations are significant, as indicated by the enhancement of *A*-coefficient which characterizes the weight of the *T*^2^ component in the electrical resistivity^14^,^15^, as well as the spin-lattice relaxation rate^23^. We speculate that the scattering lifetime is short in other Fermi surface sheets due to strong magnetic fluctuations, and hence they do not contribute significantly to MR. To accurately analyse a multiband system, an extension of Abrikosov's theory to properly include the multiband effect is desirable.

To further examine the relevance of the pocket at *Y*, we now move to CrP. In CrP, we can take (*E*_F_−*E*_c_)≈47 meV (Fig. 3e). If *γ* remains the same in CrP, *μ*_0_H*≈171 T at 0 K. Therefore, with a magnetic field of 14 T, it is not possible to have all carriers in the lowest Landau level in CrP. Furthermore, since CrP is far from the magnetic instability, multiple Fermi surface sheets with lower effective mass are contributing to the MR. Hence, the MR is quadratic.

### Absence of Shubnikov–de Haas oscillations in CrAs

We have not observed any trace of Shubnikov–de Haas (SdH) oscillations in CrAs. This might be particularly puzzling for *p*_1_ and *p*_2_, given that extra care has been taken to completely avoid the magnetic transition, so that potential spatial inhomogeniety introduced by crossing the first-order line can be ruled out. Indeed, the residual resistivity at *p*_2_ is 0.83 μΩ cm (Supplementary Note 2). However, the absence of SdH oscillations is consistent with our interpretation above, namely, that the MR is dominated by the Fermi pocket with carriers at the lowest Landau level. The SdH effect arises when successive Landau levels cross the Fermi pocket as the magnetic field is varied. In our case, since the extreme quantum limit for the pocket at *Y* is reached at 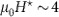 T, magnetic field higher than *μ*_0_H* will not give SdH oscillations. For magnetic field below *μ*_0_H*, SdH oscillations can in principle appear. However, the larger reduction at low field due to Dingle factor, the appearance of superconductivity below *μ*_0_*H*_c2_, and the extremely small SdH frequency all make the detection of SdH signals from this pocket extremely challenging. For other Fermi surface sheets, their short scattering lifetime due to significant magnetic fluctuations and hence their substantially weaker contributions to MR make SdH an insensitive probe to detect quantum oscillations from them.

Much can again be learnt by examining the high-pressure analogue CrP. Here the constraints stipulated above for CrAs are lifted, and SdH oscillations can readily be seen, as displayed in Fig. 1e,f. Multiple SdH frequencies are detected, with the largest frequency being ∼3.6 kT, signifying the multiband, high carrier density nature of CrP (and CrAs). In addition, the lowest frequency detected is ∼210 T (Fig. 1f). The Landau level index *n*≈*F*/(*μ*_0_*H*), where *F* is the quantum oscillation frequency. Therefore, to enter the lowest Landau level (*n*<1) the applied field *μ*_0_H* is around *F*. A satisfying agreement between the detected SdH frequency of 210 T and *μ*_0_H*≈171 T estimated in the preceeding section is noted.

### *H*–*T* scaling in CrAs under pressure

**Figure 4:**
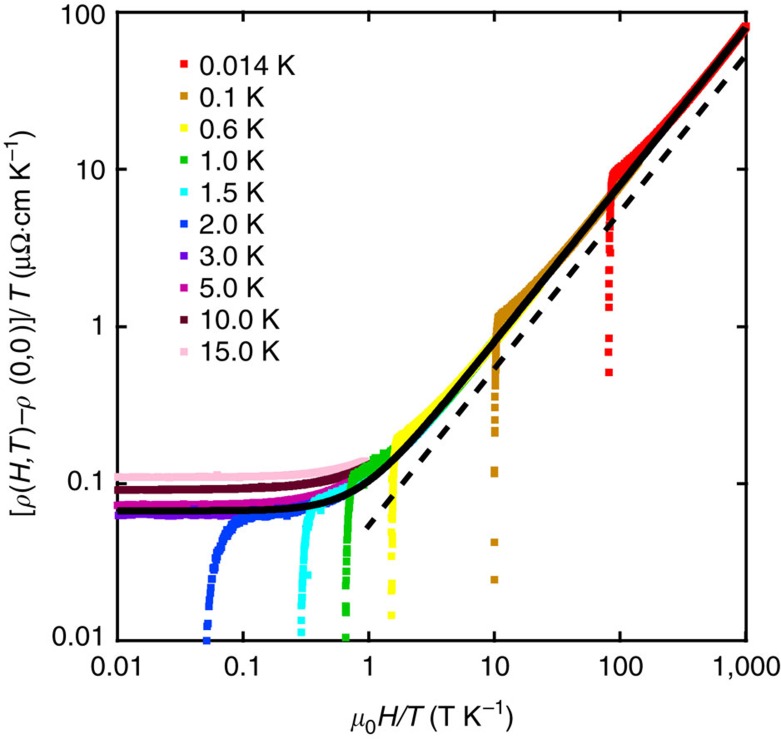
*H*–*T* scaling in CrAs at 13.1 kbar. The magnetoresistance replotted as [*ρ*(*H*, *T*)−*ρ*(0, 0)]/*T* against *μ*_0_*H*/*T*. The data below 3.0 K can all be described by a single curve *A*(1+*Bx*^2^)^0.5^ (thick solid curve), which has the same functional form as equation (3). Note that the axes are on logarithmic scales, and the dashed line indicates a unity slope.

Recently, quasilinear MR has been observed in BaFe_2_(As_1−*x*_P_*x*_)_2_ in the vicinity of the quantum critical concentration *x*_c_≈0.3 (ref. 8). Near *x*_c_, a linear *T*-dependence of electrical resistivity was reported. Incidentally, the MR is also linear in field, which led to the proposal that the resistivity would be proportional to a new energy scale that is a quadrature sum of the temperature and applied field, that is,


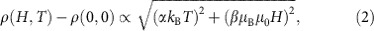


or equivalently,


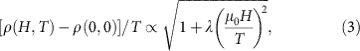


where *α*, *β* and *λ* are numerical parameters. Hence, the resistivity traces plotted as [*ρ*(*H*, *T*)−*ρ*(0, 0)]/*T* versus *μ*_0_*H*/*T* will all lie on a universal curve. In Fig. 4, we replot our data at *p*_2_=13.1 kbar on the axes of [*ρ*(*H*, *T*)−*ρ*(0, 0)]/*T* and *μ*_0_*H*/*T*. All the normal state data below 3 K indeed fall onto the same curve, except for the datapoints just above *H*_c2_. Note that both axes are on logarithmic scales. Therefore, an excellent agreement is found over five orders of magnitude in *μ*_0_*H*/*T*. Similar behaviour is found for *p*_1_ and *p*_5_ (Supplementary Note 2). On the contrary, a severe violation of Kohler's rule is found for the same data sets (Supplementary Note 3). These observations imply that the low temperature *ρ*(*T*) in the zero-field limit is linear in *T* over a wide pressure range, even though the system is tuned to a ground state that is far away from magnetism. This is reminiscent of the observation in overdoped cuprates^31^ and highlights the importance of the proximity to magnetic instability which renormalizes the effective masses of the massive bands, thereby allowing a clear dominance of high mobility fermions on magnetotransport properties. Nontrivial band crossings protected by nonsymmorphic symmetry have been attracting attention recently^28^,^32^,^33^; in the case of CrAs, it gives rise to the small Fermi pocket that allows a clear manifestation of the physics at the extreme quantum limit. Such intriguing band structure may also give rise to exotic superconducting properties as recently illustrated in UPt_3_ (ref. 34). Future studies on the influence of the nonsymmorphic symmetry will be an important theme.

## Conclusions

The subject of linear MR has a long history and it continues to attract fresh theoretical efforts (for example, refs 35, 36, 37), it is therefore possible that an alternative explanation can be found for our data. In three-dimensional Dirac semimetal Cd_3_As_2_, linear MR was attributed to mobility fluctuations in ref. 6, while alternative views were proposed for the same material^4^,^35^. In Cd_3_As_2_, Kohler's rule holds extremely well^6^, in stark contrast to pressurized CrAs. These highlight the intense interest in unraveling the underlying mechanisms giving rise to linear MR. Nevertheless, our high quality and transparent MR data, excellent *H*–*T* scaling, as well as the violation of Kohler's scaling in pressurized CrAs will provide useful constraints for a critical examination of various models.

## Methods

### Single-crystal growth

Single crystals of CrAs were grown with the Sn-flux method from a starting composition of Cr:As:Sn=1:1:10 (ref. 14). Single crystals of CrP were prepared by chemical vapour transport method^38^. The powdered polycrystalline sample of CrP was sealed in a silica tube with iodine under vacuum. The charge zone was kept at 900 °C and the growth zone at 800 °C for 2 weeks.

### Electrical resistance measured under extreme conditions

The high-pressure environment was provided by a piston-cylinder clamp cell made of MP35N alloys. Glycerin was used as the pressure medium and the pressure values were estimated by the zero-field *T*_c_ of a Pb manometer placed near the sample. The pressure cell was cooled down to about 15 mK in a dilution refrigerator (BlueFors) equipped with a 14 T superconducting magnet (American Magnetic Inc.) for all pressure points except *p*_4_=12.5 kbar, which was examined with a Physical Property Measurement System (Quantum Design), with a base temperature of 1.8 K. The MR was measured with a conventional four-probe method with the magnetic field parallel to the *c* axis and the current along *a* axis.

### Band structure calculations

Band structure calculations were performed with the all-electron full-potential linearized augmented plane-wave code WIEN2k (ref. 39). The generalized gradient approximation of Perdew, Burke and Ernzerhof^40^ was employed for the exchange-correlation potential. Experimental lattice constants^24^,^41^ were used in the calculation and internal structure optimization was performed. The muffin-tin radii were set to 1.96 a.u. for the P atoms and 2.24 a.u. for the Cr and As atoms. 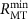
*K*_max_=8 and a *k*-point mesh of 5,000 in the first Brillouin zone were used.

### Data availability

The data used for supporting the findings of this study are available from the corresponding authors on request.

## Additional information

**How to cite this article:** Niu, Q. *et al*. Quasilinear quantum magnetoresistance in pressure-induced nonsymmorphic superconductor chromium arsenide. *Nat. Commun.* **8,** 15358 doi: 10.1038/ncomms15358 (2017).

**Publisher's note**: Springer Nature remains neutral with regard to jurisdictional claims in published maps and institutional affiliations.

## Supplementary Material

Supplementary Information Supplementary Figures, Supplementary Notes, Supplementary Table and Supplementary References
