# Supplementary material for: Quasilinear quantum magnetoresistance in pressure-induced nonsymmorphic superconductor chromium arsenide
Source: Nat Commun. 2017 Jun 5;8:15358. doi: 10.1038/ncomms15358 (PMC5465317; doi:10.1038/ncomms15358)
Supplement: Supplementary Information — Supplementary Figures, Supplementary Notes, Supplementary Table and Supplementary References [file ncomms15358-s1.pdf]

Supplementary Information

Supplementary Note 1: Experimental verification of the calculated hybrid band structure around the Y point

In Figure 3 of the main text, we show the calculated band structure of CrAs at 14.3 kbar and CrP at ambient pressure. We focus on the band structure near the Y point, where a small Fermi surface pocket is located. According to Figure 3, the dispersion is linear along Γ–Y direction and parabolic along Y–S ($\parallel k_x$) direction, which is analogous to the scenario of the semi-Dirac point discussed in the literature [1, 2], except that the parabolic branch with a negative mass does not exist in our case. The dispersion relation around the Y point near E_F can be written as

$$\varepsilon = \hbar c\sqrt{k_y^2 + \delta^2 k_z^2} + \frac{\hbar^2 k_x^2}{2m}. \quad (1)$$

Note that (k_x, k_y, k_z) is a wavevector measured from Y. For the semiclassical approach, the areal quantization condition in a field $\mu_0 H$ ($\parallel k_z$) has the form of

$$S(\varepsilon) = 2\pi(n+\gamma)e\mu_0 H/\hbar, \quad (2)$$

where $\gamma \in [0, 1]$ is the phase factor which can not be determined by the semiclassical treatment. The area of an orbit at k_z =0 is

$$S(\varepsilon) = \oint k_y dk_x = 4\int_0^{\frac{\sqrt{2m\varepsilon}}{\hbar}} k_y dk_x = \frac{8}{3}\frac{\sqrt{2m}}{\hbar^2 c}\varepsilon^{3/2}. \quad (3)$$

Thus, the Landau levels at $k_z = 0$ are

$$\varepsilon_n = \left[\frac{3\hbar c}{8\sqrt{2m}} 2\pi(n+\gamma)e\mu_0 H\right]^{2/3}. \quad (4)$$

Therefore, the energy spacing between the $n = 0$ and $n = 1$ Landau levels is

$$\Delta_1 = \varepsilon_1 - \varepsilon_0 = \left[\frac{3\hbar c}{8\sqrt{2m}} 2\pi e\mu_0 H\right]^{2/3} f(\gamma), \quad (5)$$

where $f(\gamma) = (1+\gamma)^{2/3} - \gamma^{2/3}$.

According to Abrikosov's theory, when all the carriers occupy only the zeroth Landau Level (the 'extreme quantum limit'), the MR will linearly depend on the magnetic field [3]. To realize this situation, the Landau level splitting between the zeroth and the first Landau level must be larger than the Fermi energy of the system, *i.e.* $\Delta_1 > E_\mathrm{F} - E_\mathrm{c}$, at $T = 0$ K. At finite temperature, the effect of thermal fluctuation must be taken into account [4]. These considerations lead to equation (1) in the main text, *i.e.*

$$\mu_0 H^* = \frac{8\sqrt{2m}}{6\pi e\hbar c}[f(\gamma)]^{-3/2}((E_\mathrm{F} - E_\mathrm{c}) + k_\mathrm{B} T)^{3/2}, \quad (6)$$

where $(E_\mathrm{F} - E_\mathrm{c})$ is the energy difference between the Fermi energy and the crossing point.

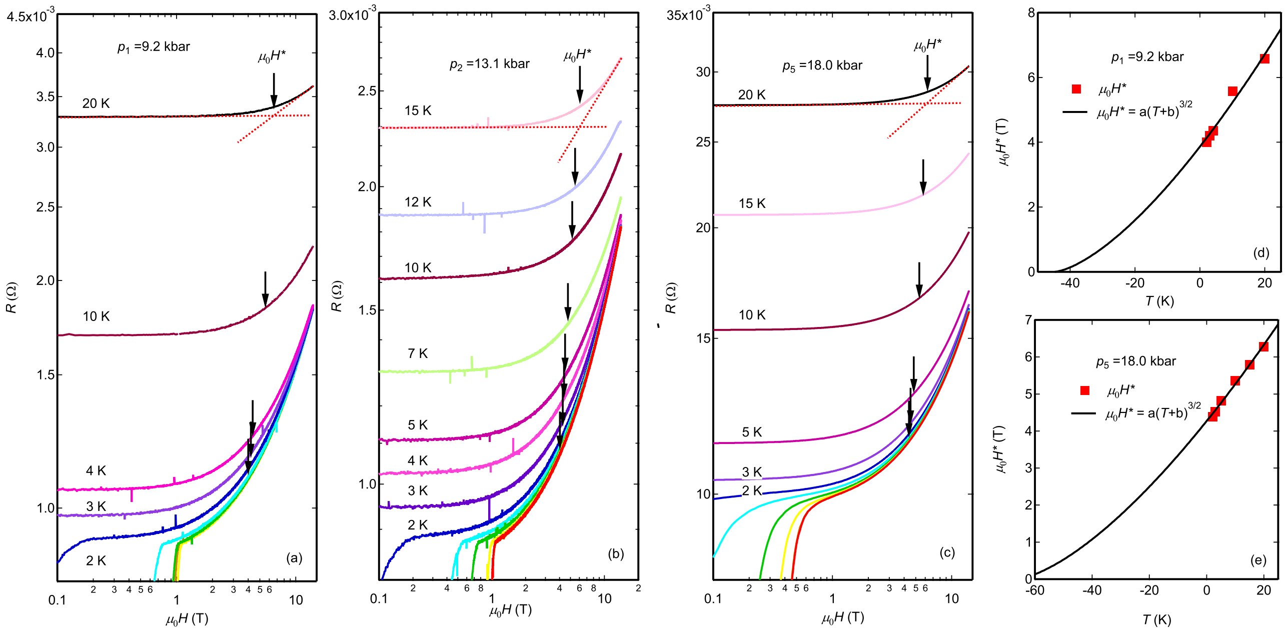

Supplementary Figure 1. **$\mu_0 H^*$ of the pressurised CrAs.** (a)–(c) Determination of $\mu_0 H^*$ at p_1=9.2 kbar, p_2=13.1 kbar, and p_5=18.0 kbar. We define $\mu_0 H^*$ at a given temperature as the intersection of two straight dashed lines as shown in the figures. The black arrows mark the values of $\mu_0 H^*$. (d)–(e) $\mu_0 H^*(T)$ fitted with Supplementary Equation 6 at p_1=9.2 kbar, and p_5=18.0 kbar.

Similar to the presentation in the main text, where we show the experimental determination of $\mu_0 H^*$ using the dataset at p_2=13.1 kbar, Supplementary Figure 1 (a)–(c) show the determination of $\mu_0 H^*$ for p_1=9.2 kbar, p_2=13.1 kbar, and p_5=18.0 kbar. We remind the readers that the resistances are plotted against the magnetic field on log scales, and $\mu_0 H^*$ at a given temperature is determined using the intersection of two straight dashed lines (see arrows in Supplementary Figure 1 (a)–(c)). Supplementary Figure 1 (d)–(e) show the corresponding $\mu_0 H^*$ as a function of temperature. The solid lines are the fitting curves with formula

$$\mu_0 H^* = a \cdot (T + b)^{3/2}, \tag{7}$$

where

$$a = \frac{8\sqrt{2m}}{6\pi e\hbar c}[f(\gamma)]^{-3/2} k_{\rm B}^{3/2}, b = \frac{(E_{\rm F} - E_{\rm c})}{k_{\rm B}}. \tag{8}$$

The fitting parameter b immediately gives $(E_{\rm F} - E_{\rm c})$, the results are summarized in Supplementary Table . The other fitting parameter a is related to the mass m, the slope of the dispersion c and $f(\gamma)$. To estimate m and c, we need to consider another constraint based on the anisotropy of the Fermi surface on the $k_x - k_y$ plane.

As mentioned in the main text, we can define an anisotropic factor $\alpha = k_{\rm F}^x / k_{\rm F}^y$. Furthermore, we have the following

$$E_{\rm F} - E_{\rm c} = \hbar c k_{\rm F}^y = \hbar c k_{\rm F}^x / \alpha \tag{9}$$

$$E_{\rm F} - E_{\rm c} = \frac{\hbar^2 k_{\rm F}^{x\,2}}{2m} \tag{10}$$

From Supplementary Equation (9) and (10), we have the relation quoted in the main text

$$m = \frac{\alpha^2}{2c^2}(E_{\rm F} - E_{\rm c}) \tag{11}$$

Combine Supplementary Equation (8) and (11), we have

$$c(\gamma) = c(0)[f(\gamma)]^{-3/4}, \text{where } c(0) = k_{\rm B}\sqrt{\frac{4\sqrt{b}}{3\pi e\hbar a}}\sqrt{\alpha} \tag{12}$$

$$m(\gamma) = m(0)[f(\gamma)]^{3/2}, \text{where } m(0) = \frac{3\pi e\hbar a\sqrt{b}}{8k_{\rm B}}\alpha \tag{13}$$

Using the band structure displayed in Figure 3(b) of the main text, we adjust the Fermi energy to match the experimentally extracted $(E_{\rm F} - E_{\rm c})$ from the fitting parameter b for a given pressure, while keeping the band structure intact (rigid band shift). This allows us to calculate m and c for p_1=9.2 kbar, p_2=13.1 kbar, and p_5=18.0 kbar, with the results tabulated in Supplementary Table .

pressure (kbar)	$E_{\rm F} - E_{\rm c}$ (meV)	α	$c(0)$ ($\times 10^4$ m/s)	$c(1)$ ($\times 10^4$ m/s)	$m(0)(m_{\rm e})$	$m(1)(m_{\rm e})$
9.2	3.9	4.62	10.8	17.1	0.63	0.28
13.1	3.7	4.74	10.5	15.6	0.66	0.30
18.0	5.7	3.80	13.0	19.3	0.43	0.20

Supplementary Table 1. **The experimentally extracted parameters for the hybrid band structure at the Y point for different pressures.** $(E_{\rm F} - E_{\rm c})$ is the energy separation between the crossing point and the Fermi energy, α is the anisotropic factor, c and m parametrise the linear and the parabolic energy-momentum dispersion relations, respectively. $m_{\rm e}$ is the bare electron mass.

Supplementary Note 2: H–T scaling

To get the H–T scaling plot as shown in Fig. 4 of the main text, we need to estimate $\rho(0,0)$. We use linear extrapolation of the base temperature $\rho(\mu_0 H)$ curve above $\mu_0 H^*$ and get $\rho(0,0) \approx 0.83$ $\mu\Omega$.cm, as shown in Supplementary Figure 2 (a). For the other two pressure values, *i.e.*, p_1=9.2 kbar, and p_5=18.0 kbar, we follow the same procedure. In Supplementary Figure 2 (b) and (c), we plot $[\rho(\mu_0 H, T) - \rho(0,0)]/T$ versus $\mu_0 H/T$ on log scales for the other two pressures.

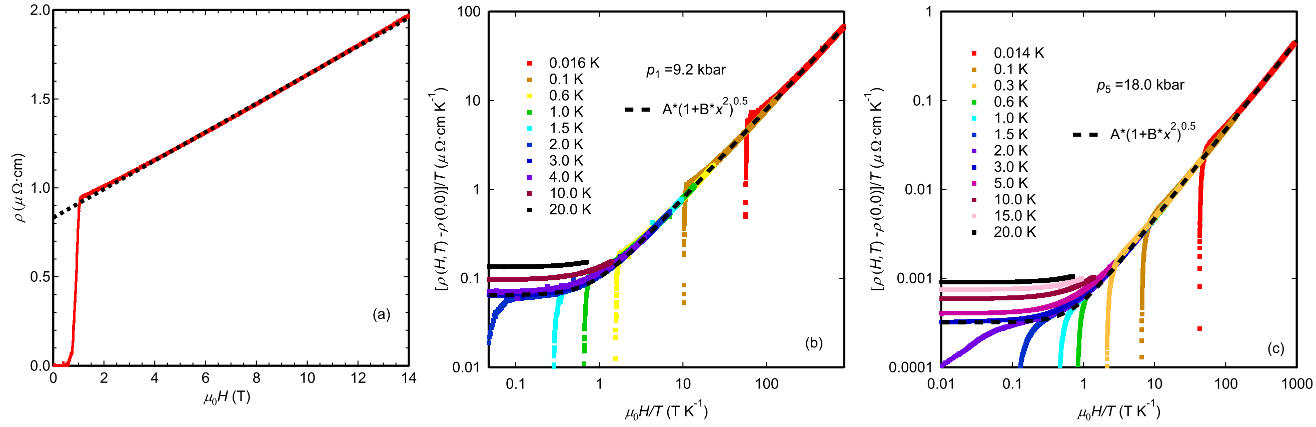

Supplementary Figure 2. **Construction of the** H–T **scaling.** (a) Linear extrapolation of $\rho(\mu_0 H)$ curve at p_2=13.1 kbar; (b) and (c) $H - T$ scaling at p_1=9.2 kbar, and p_5=18.0 kbar.

Supplementary Note 3: Violation of Kohler's rule

To contrast with the H–T scaling in the preceding section, we construct the Kohler plot for the same datasets. As shown in Supplementary Figure 3, a serious violation of Kohler's rule is observed for all three pressures. In fact, the new H–T scaling is not compatible with Kohler's rule [5]. Hence, we do not expect Kohler's rule to hold given the applicability of H–T scaling.

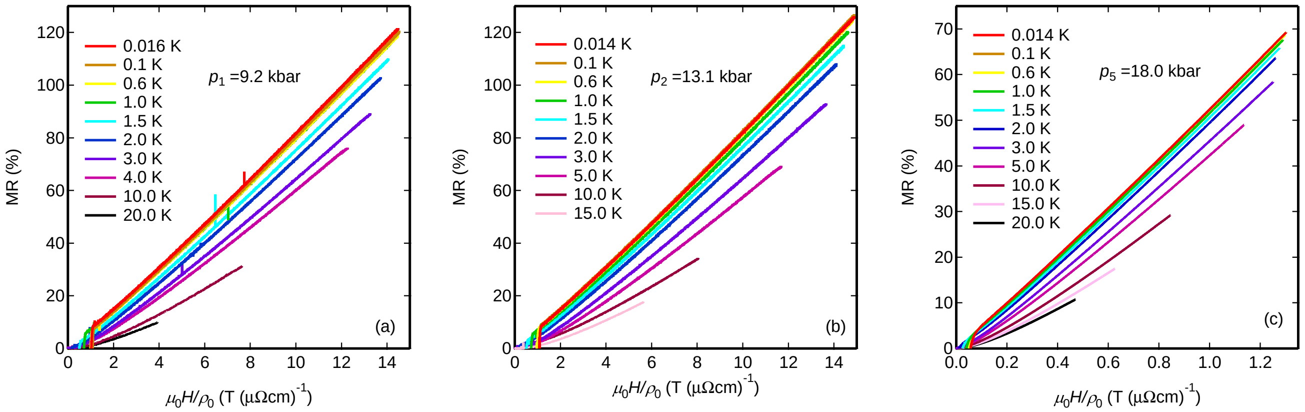

Supplementary Figure 3. **Violation of Kohler's scaling in pressurised CrAs.** (a)–(c) Kohler's plot at p_1=9.2 kbar, p_2=13.1 kbar, and p_5=18.0 kbar.

Supplementary Note 4: Symmetry protection of the band crossing

In this section, we provide a detailed analysis based on the nonsymmorphic symmetry to prove the band crossing on the Y–S line. On the Y–S line which lies on the face of the first Brillouin zone, flipped wave vectors $m_y\boldsymbol{k} = (k_x, -k_y, k_z)$ and $m_z\boldsymbol{k} = (k_x, k_y, -k_z)$ are equivalent to $\boldsymbol{k} = (k_x, k_y, k_z)$, because $k_z = 0$ and $m_y\boldsymbol{k} = \boldsymbol{k}+\boldsymbol{G}$ with $\boldsymbol{G}$ being a reciprocal lattice vector. Thus, the k-group involves the glide symmetry G^z and the mirror symmetry M^y, which are symmetry elements of the space group $Pnma$. The glide symmetry $G^z = \{M^z|\frac{x}{2}\}$ is a combined symmetry of mirror reflection M^z and half translation along the x-axis. Thus, the nonsymmorphic symmetry is preverved on the Y–S line.

Because four Cr atoms are involved in the unit cell, the symmetry operators are represented by three sets of Pauli matrices, s_i, σ_i, and τ_i ($i = 0, 1, 2, 3$). While s_i acts on the spin space, σ_i and τ_i are operators in the sublattice space. Two inequivalent sites in zigzag chains are operated by σ_i, and two inequivalent zigzag chains are operated by τ_i (see Supplementary Figure 4).

The symmetry operators are represented by

$$\hat{M}^y(\boldsymbol{k}) = is_y \otimes \sigma_0 \otimes \begin{pmatrix} 1 & 0 \\ 0 & e^{ik_y} \end{pmatrix}_\tau , \tag{14}$$

$$\hat{G}^z(\boldsymbol{k}) = is_z \otimes \begin{pmatrix} 0 & e^{-ik_x} \\ 1 & 0 \end{pmatrix}_\sigma \begin{pmatrix} 1 & 0 \\ 0 & e^{-ik_z} \end{pmatrix}_\tau , \tag{15}$$

$$\hat{I}\hat{T} = is_y \otimes \sigma_x \otimes \tau_x K, \tag{16}$$

where $\hat{I}\hat{T}$ is inversion-time-reversal symmetry preserving all the wavevectors $\boldsymbol{k}$, and K is the complex conjugate operator. In the following, we consider reduced operators on the Y–S line,

$$\hat{M}^y(\boldsymbol{k}) = is_y \otimes \sigma_0 \otimes \tau_z, \tag{17}$$

$$\hat{G}^z(\boldsymbol{k}) = is_z \otimes \begin{pmatrix} 0 & e^{-ik_x} \\ 1 & 0 \end{pmatrix}_\sigma \otimes \tau_0. \tag{18}$$

Because the single electron part of the Hamiltonian $\hat{H}_0(\boldsymbol{k})$ commutes with the mirror reflection operator $\hat{M}^y(\boldsymbol{k})$ on the Y–S line, the Hamiltonian is block-diagonalized on the basis spanned by eigenstates of $\hat{M}^y(\boldsymbol{k})$. From the

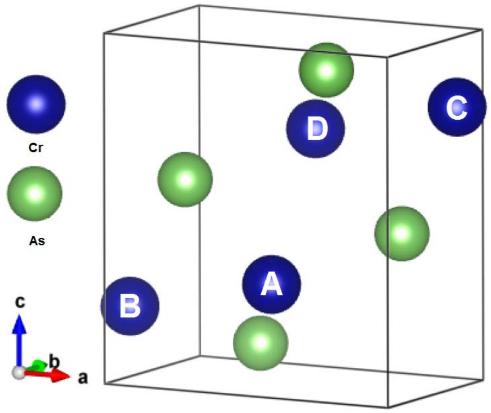

Supplementary Figure 4. Crystal structure of CrAs. Cr atoms in the unit cell are labeled by A, B, C, and D. The Pauli matrix σ_i acts on the sublattice space spanned by A and B (and also by C and D). On the other hand, τ_i is the operator on the space spanned by A and C (and also by B and D).

relation $\left[\hat{M}^y(\boldsymbol{k})\right]^2 = -1$ we obtain mirror eigenvalues, $\lambda = \pm i$. The Hamiltonian is decomposed into the two mirror subsectors,

$$\hat{H}_0(\boldsymbol{k}) = \hat{H}_i(\boldsymbol{k}) \oplus \hat{H}_{-i}(\boldsymbol{k}). \tag{19}$$

We can show that the inversion-time-reversal symmetry is preserved in each mirror subsector by using the anti-commutation relation, $\left\{\hat{M}^y(\boldsymbol{k}), \hat{I}\hat{T}\right\} = 0$. Then, the inversion-time-reversal symmetry ensures Kramers degeneracy in each subsector as the anti-unitary operator satisfies the usual relation, $\left(\hat{I}\hat{T}\right)^2 = -1$. We label Kramers pairs in the mirror subsector $|\alpha, \pm\rangle_{\pm i}$.

Now we consider the glide symmetry. Since the glide symmetry is preserved, $\hat{G}^z(\boldsymbol{k})|\alpha, \pm\rangle_{\pm i}$ are eigenstates of the Hamiltonian with the same energy as $|\alpha, \pm\rangle_{\pm i}$. On the other hand, the mirror eigenvalue of $\hat{G}^z(\boldsymbol{k})|\alpha, \pm\rangle_{\pm i}$ is $\mp i$, as it is proved by $\left\{\hat{G}^z(\boldsymbol{k}), \hat{M}^y(\boldsymbol{k})\right\} = 0$,

$$\hat{M}^y(\boldsymbol{k})\left(\hat{G}^z(\boldsymbol{k})|\alpha, \pm\rangle_{\pm i}\right) = -\hat{G}^z(\boldsymbol{k})\hat{M}^y(\boldsymbol{k})|\alpha, \pm\rangle_{\pm i} = -\hat{G}^z(\boldsymbol{k})\left(\pm i|\alpha, \pm\rangle_{\pm i}\right) = \mp i\ \hat{G}^z(\boldsymbol{k})|\alpha, \pm\rangle_{\pm i}. \tag{20}$$

Therefore, $\hat{G}^z(\boldsymbol{k})|\alpha, \pm\rangle_{\pm i}$ are eigenstates in the mirror subsector $\mp i$. This ensures that four eigenstates, $|\alpha, \pm\rangle_{\pm i}$ and $\hat{G}^z(\boldsymbol{k})|\alpha, \pm\rangle_{\pm i}$, have the same energy and orthogonal to each other. The four-fold degeneracy in the single-particle electron states requires additional degeneracy in the band structure on the Y–S line, in addition to the usual spin degeneracy protected by the inversion-time-reversal symmetry. Thus, the band crossing on the Y–S line is protected by symmetry, as demonstrated by the calculated band structure in Fig. 3 of the main text.

It should be stressed that the nonsymmorphic symmetry plays an essential role for the band crossing. The square of the two-fold glide or screw operation is nothing but the translation of the unit cell, which is represented by $e^{-i\boldsymbol{k}\cdot\boldsymbol{\tau}}$ with τ being a lattice vector. Thus, the nonsymmorphic operators must be momentum-dependent. This will affect the (anti)commutation relations between symmetry operators, when one moves from the Brillouin zone face to the Brillouin zone center. For example, the commutation relation $\left[\hat{M}^y(\boldsymbol{k}), \hat{I}\hat{T}\right] = 0$ is satisfied on the Γ–X line, instead of the anticommutation relation used in the proof above. Therefore, the band crossing does not occur on the Γ-X line. In this case, the mirror reflection operator is momentum-dependent because the mirror symmetry is a combined symmetry of the inversion symmetry I and the nonsymmorphic screw symmetry $S^y_\pi = \left\{R^y_\pi | \frac{y}{2}\right\}$.

Supplementary references

[1] Dietl, P., Piéchon, F., Montambaux, G., New Magnetic Field Dependence of Landau Levels in a Graphenelike Structure. Phys. Rev. Lett. **100**, 236405 (2008).

[2] Banerjee, S., Singh, R. R. P., Pardo, V., Pickett, W. E., Tight-Binding Modeling and Low-Energy Behavior of the Semi-Dirac Point. Phys. Rev. Lett. **103**, 016402 (2009).

[3] Abrikosov, A. A., Quantum magnetoresistance. Phys. Rev. B **58**, 2788–2794 (1998).

[4] Huynh, K. K.,Tanabe, Y.,Tanigaki, K., Both electron and hole Dirac cone states in $Ba(FeAs)_2$ confirmed by magnetoresistance. Phys. Rev. Lett. **106**, 217004 (2011).

[5] Hayes, I. M., McDonald, R. D., Breznay, N. P., Helm, T., Moll, P. J. W., Wartenbe, M., Shekhter, A., Analytis, J. G., Scaling between magnetic field and temperature in the high-temperature superconductor $BaFe_2(As_{1-x}P_x)_2$. Nature Phys. **12**, 916-919 (2016).
